# New Publications

**Published:** 1896-03

**Authors:** 


### NEW PUBLICATIONS.

THE VETERINARY HERALD, a students' journal, makes its initial bow to the student fraternity with the January issue. It is edited and published by the students of the Chicago Veterinary College and is the first publication of its kind in this country. While its field is necessarily limited, it has great opportunities to do much good, and we wish for it a successful career.

The report of the Massachusetts Cattle Commission in book form has reached us and sheds a deal of light upon the subject of tuberculosis in all its aspects, and will prove a valuable book of reference for all interested in the subject, as well as a valuable guide for all those who contemplate legislation, State or local, designed to deal with this now all-important question.

